# Report on Urinary Antiseptics

**Published:** 1914-01

**Authors:** 


Report on Urinary Antiseptics. Anson Jordan, *British Med. Jour.*, Sept. 13, 1913. Jordan reports the result of his investigation under the auspices of the British Medical Association. The work was experimental, the effect of chemical reaction and of various drugs upon urinary putrefaction and upon the growth of selected microörganism in the urine being observed *in vitro*. The following practical conclusions are drawn:

1. The acidity of the urine is readily increased to an extent of more than double the normal by acid sodium phosphate, ($NaH_2PO_4$, average dosage gr. XV, t.i.d.) and to a less extent by benzoates. With large doses of citrates it is easily rendered alkaline (*e. g.*, sodium citrate gr. XXX, t.i.d.)

2. Putrefaction of the urine and the growth of the staphylococcus is aided by alkalinity and delayed by acidity in proportion to the amount thereof. The reverse is true with B. coli, but only to a small extent, its growth in both acid and alkaline urines being quite luxuriant.

3. Hexamethylenetetramine (urotropin) is not itself antiseptic, but acts by producing formaldehyde in the urine. This takes the place only in acid urine, the yield of formaldehyde, and therefore the degree of antiseptic power, being proportionate to acidity. This drug is by far the most efficient of all the urinary antiseptics.

4. Helmitol, citramine, hetraline and cystopurin, though they all yield formaldehyde in alkaline media in the test tube, behave precisely like urotropine in the urine, having no antiseptic power in alkaline urine.

5. Sandalwood oil, though not an efficient general urinary antiseptic, seems to have a specific action on the staphylococcus, which possibly accounts for its reputed favorable action in gonorrhea.

6. Benzoic and salicylic acids are fairly efficient urinary antiseptics, but of little use in alkaline urine.

7. Boric acid acts efficiently; its action being unaffected by alkalinity. It is the most efficient drug in alkeline urine we possess.

8. Uva ursi is quite a good antiseptic. Its action is certainly not due chiefly to the arbutin it contains.

